# Discovery of clinical isolation of drug-resistant *Klebsiella pneumoniae* with overexpression of OqxB efflux pump as the decisive drug resistance factor

**DOI:** 10.1128/spectrum.00122-24

**Published:** 2024-08-16

**Authors:** Ping Tian, Ming-Juan Guo, Qing-Qing Li, Xu-Feng Li, Xiao-Qiang Liu, Qin-Xiang Kong, Hui Zhang, Yi Yang, Yan-Yan Liu, Liang Yu, Jia-Bin Li, Ya-Sheng Li

**Affiliations:** 1 Department of Infectious Diseases, The First Affiliated Hospital of Anhui Medical University, Hefei, China; 2 Anhui Province Key Laboratory of Infectious Diseases, Anhui Medical University, Hefei, China; 3 Anhui Center for Surveillance of Bacterial Resistance, The First Affiliated Hospital of Anhui Medical University, Hefei, China; 4 Institute of Bacterial Resistance, Anhui Medical University, Hefei, China; 5 Department of Hepatology, The First Affiliated Hospital of Jilin University, Changchun, China; 6 Department of Infectious Diseases, Chaohu Hospital of Anhui Medical University, Hefei, China; Université Paris-Saclay, Saint Aubin, France

**Keywords:** *Klebsiella pneumoniae*, quinolones-resistant, efflux pump, OqxB, PaβN

## Abstract

Background emergence of multidrug-resistant (MDR) bacterial strains is a public health concern that threatens global and regional security. Efflux pump-overexpressing MDR strains from clinical isolates are the best subjects for studying the mechanisms of MDR caused by bacterial efflux pumps. A *Klebsiella pneumoniae* strain overexpressing the OqxB-only efflux pump was screened from a clinical strain library to explore reverse OqxB-mediated bacterial resistance strategies. We identified non-repetitive clinical isolated *K. pneumoniae* strains using a matrix-assisted laser desorption/ionization time-of-flight (TOF) mass spectrometry clinical TOF-II (Clin-TOF-II) and susceptibility test screening against levofloxacin and ciprofloxacin. And the polymorphism analysis was conducted using pulsed-field gel electrophoresis. Efflux pump function of resistant strains is obtained by combined drug sensitivity test of phenylalanine-arginine beta-naphthylamide (PaβN, an efflux pump inhibitor) and detection with ethidium bromide as an indicator. The quantitative reverse transcription PCR was performed to assess whether the *oqxB* gene was overexpressed in *K. pneumoniae* isolates. Additional analyses assessed whether the *oqxB* gene was overexpressed in *K. pneumoniae* isolates and gene knockout and complementation strains were constructed. The binding mode of PaβN with OqxB was determined using molecular docking modeling. Among the clinical quinolone-resistant *K. pneumoniae* strains, one mediates resistance almost exclusively through the overexpression of the resistance–nodulation–division efflux pump, OqxB. Crystal structure of OqxB has been reported recently by N. Bharatham, P. Bhowmik, M. Aoki, U. Okada et al. (Nat Commun 12:5400, 2021, https://doi.org/10.1038/s41467-021-25679-0). The discovery of this strain will contribute to a better understanding of the role of the OqxB transporter in *K. pneumoniae* and builds on the foundation for addressing the threat posed by quinolone resistance.

**IMPORTANCE:**

The emergence of antimicrobial resistance is a growing and significant health concern, particularly in the context of *K. pneumoniae* infections. The upregulation of efflux pump systems is a key factor that contributes to this resistance. Our results indicated that the *K. pneumoniae* strain GN 172867 exhibited a higher *oqxB* gene expression compared to the reference strain ATCC 43816. Deletion of *oqxB* led a decrease in the minimum inhibitory concentration of levofloxacin. Complementation with *oqxB* rescued antibiotic resistance in the *oqxB* mutant strain. We demonstrated that the overexpression of the OqxB efflux pump plays an important role in quinolone resistance. The discovery of strain GN 172867 will contribute to a better understanding of the role of the OqxB transporter in *K. pneumoniae* and promotes further study of antimicrobial resistance.

## INTRODUCTION

According to a recent United Nations Environment Programme report, if antibiotic resistance continues to increase, 10 million people will die each year by 2050, which is a higher mortality rate than that associated with cancer (1). The correct use of antibiotics and the development of novel antibiotics are the only ways to cope with this public health crisis. *Enterobacteriaceae*, *Acinetobacter baumannii*, and *Pseudomonas aeruginosa* were identified by the World Health Organization as the three critical priority pathogens for new antibiotic development beginning in 2017 (2, 3). However, only a few new antibiotics have been identified in recent decades (4), and it is important to continue to expand on the use of traditional antibacterial drugs (5, 6). Quinolones are widely used for anti-infective therapy owing to their unique antibacterial mechanisms; however, the emergence of widespread resistance to quinolones in clinically isolated strains has reduced their use as a first-line therapy (7). Thus, studying the mechanism of pathogen resistance to quinolones and restoring their therapeutic effects are of great significance for clinical treatment.

One of the most notorious pathogens of the *Enterobacteriaceae* family is *Klebsiella pneumoniae*, which causes meningitis, pneumonia, severe bacteremia, liver abscesses, and other invasive community-acquired infections (8). Unfortunately, resistance mechanisms such as target gene mutations, drug inactivation, decreased entry, and increased efflux have been reported in *K. pneumoniae* and can result in reduced sensitivity to quinolones (9). To date, six major families of efflux pumps have been identified in bacteria: (1) resistance–nodulation–cell division (RND) (2), ATP-binding cassette (ABC) (3), major facilitator superfamily (MFS) (4), multidrug and toxic compound extrusion (5), small multidrug resistance (SMR), and (6) proteobacterial antimicrobial compound efflux families. The RND superfamily includes OqxA, OqxB, AcrA, AcrB, TolC, and QepA, which is the primary efflux pump family that mediates antibiotic resistance in Gram-negative bacteria. KdeA is an MFS efflux pump. KpnE is an SMR efflux pump (10, 11), and OqxAB is associated with another set of clinical isolates of *K. pneumoniae* (12). Antibiotic resistance has plagued the treatment options for *K. pneumoniae* as a clinical pathogen. Therefore, it is important to explore the specific mechanism by which clinical strains tolerate quinolones to improve quinolone use and provide new treatment options for *K. pneumoniae* infection treatment strategies.

In this study, 12 clinical quinolone-resistant *K. pneumoniae* strains preserved by the Anhui Bacterial Resistance Center were used to investigate the resistance mechanisms by analyzing the expression of resistance genes. One strain (GN 172867), which mediated resistance almost exclusively through the OqxB efflux pump overexpression, was identified. OqxB is an RND efflux pump, whose crystal structure was recently reported by Bharatham et al. (13).We believe that OqxB-overexpressing strains derived from clinical isolates are the most important object to investigate OqxB-mediated multidrug resistance. We used minimum inhibitory concentration (MIC) assays, quantitative real-time reverse-transcription PCR (qRT-PCR), and ethidium bromide (EtBr)-agar cartwheel assays, deletion and complementation of *oqxB*, and molecular modeling docking to characterize OqxB-overexpressing strains derived from clinical isolates and investigate OqxB-mediated multidrug resistance. We believe that this work will contribute to a better understanding of the role of the OqxB transporter in *K. pneumoniae* and provide a new platform for addressing quinolone resistance in bacteria.

## MATERIALS AND METHODS

### Bacterial isolates and antimicrobials

Twelve non-repetitive *K. pneumoniae* strains were identified between January 2017 and December 2020 using qualifying data from clinical samples maintained at the Anhui Center for Surveillance of Bacterial Resistance (14) using a matrix-assisted laser desorption/ionization time-of-flight mass spectrometry (MALDI-TOF MS) clinical TOF-II (Clin-TOF-II) Automated Microbiology System (BIOYONG, China) (15, 16). All isolates were cultivated on Muller–Hinton Agar (MHA; Sigma-Aldrich, USA) at 37°C and stored in Muller–Hinton Broth (MHB; Sigma-Aldrich, USA) with 50% glycerol in cryovials for stock at −80°C. The wild-type standard strains were *Escherichia coli* ATCC 8739 and *K. pneumoniae* ATCC 43816 that were used as controls.

All the antibiotics, phenylalanine-arginine-β-naphthylamide (PaβN) (17), verapamil, and EtBr were purchased from Sigma-Aldrich (St. Louis, MO, USA). Prior to usage, each sample was stored at −20°C for less than 2 weeks.

### Pulsed-ﬁeld gel electrophoresis

Pulsed-field gel electrophoresis (PFGE) was applied to each isolated strain. The restriction endonuclease, *XbaI* (TaKaRa, Lot# AIF2232A), was used for genomic DNA digestion, and *Salmonella* H9812 served as the DNA size marker for PFGE electrophoresis. Cells of each strain were prepared using gelatinization, enzymatic digestion, and electrophoresis. Then, a tree diagram was created using BioNumerics software to process the PFGE images. The strains in the similarity analysis matrix had similarity coefficients (>80%) and belonged to the same PFGE class (18).

### Gene expression analysis by quantitative PCR

The expression of efflux pump genes was assessed using the qRT-PCR (19) with the primers listed in Table S2. After an overnight incubation period, all cultures were diluted 100 times in MHB for late log phase of growth [optical density at 600 nm (OD_600_) = 0.6] at 37°C and 220 rpm. RNA was extracted using an RNeasy Kit (Qiagen, Hilden, Germany) according to the manufacturer’s instructions, and copy DNA (cDNA) was generated for reverse transcription using a PrimeScript cDNA synthesis kit (TaKaRa, Kyoto, Japan). A three-step Real-Time PCR System (Light Cycler 96; Roche, Basel, Switzerland) was used for qRT-PCR to measure the following genes: *acrA*, *acrB*, *tolC*, *oqxA*, *oqxB*, *luxS*, *qepA*, *kdeA*, *kpnE*, *iucA*, *VIM*, *NDM*, *OXA-48*, *CTX-M-2*, *SHV*, *TEM*, *qnrA*, *qnrB*, *qnrS*, *magA*, *aer*, *iroB*, *irp2*, *entB*, and *kfu* using the PrimeScript RT Master Mix (TaKaRa). Relative quantification of the target gene expression was performed by normalizing target gene expression to that of an endogenous reference (*rrsE*) that encodes the 16S ribosomal RNA-coding gene (20–22). A Light Cycler 96 was used for data analysis. The expression ratios were calculated using the 2^−ΔΔCT^ method and presented as fold change values. The reference strain for the gene expression analysis was *K. pneumoniae* ATCC 43816. Each reaction was performed in triplicate.

### Antimicrobial susceptibility testing

Susceptibility testing was performed using the broth dilution method according to the guidelines of the Clinical and Laboratory Standards Institute (23). The inoculum density of the isolated colonies was standardized to 0.5 McFarland standard turbidity (~1.5 × 10^8^ CFU/mL) using phosphate-buffered saline, diluted with Mueller–Hinton broth and adjusted to a bacterial concentration of 1 × 10^6^ CFU/mL. Antibacterial drugs were added to the cultures at final concentrations ranging from 1 to 512 mg/L. MHB medium served as the negative control, and a drug-free well was designated as the growth control. After 16–18 h of incubation at 37℃, the OD_600_ was measured using a microplate reader, and visual reading was also used to calculate the MICs. Following microplate scanning, the lowest concentration of the drug (OD_600_ < 0.1) was recorded as the MIC (24). The MICs for the different strains were measured in quadruplicate. The strain used for quality control was ATCC 43816.

### Efflux pump inhibition test

Verapamil and PaβN are two efflux pump inhibitors (EPIs) and were used to identify efflux pump activity in multidrug-resistant (MDR) *K. pneumoniae* isolates. The MICs of several antibiotics were ascertained using the broth dilution method with and without 50-mg/L PaβN or verapamil. According to MIC reduction with verapamil or PaβN, the key efflux pump was confirmed (25). Three separate EPI experiments were conducted.

### Growth curve determination

Briefly, the bacterial cultures were diluted overnight in MHB medium until their OD_600_ was 0.05. Then, a 100-µL volume of the bacteria solution was added to each well, either with or without a quinolone antibiotic, PaβN, or both. There was only one growth medium in each of the four wells. The cultures were cultivated at 37°C and 886.9 rpm in an automated microplate reader (Tecan, Switzerland) until they reached the stationary phase. Every 60 min, OD_600_ measurements were recorded while continuously shaking the cultures (26).

### EtBr-agar cartwheel method

Using agar as the substrate, this method uses the fluorochrome EtBr to detect the presence of an overexpressed efflux system of the test strain compared to that of the endogenous efflux system of the equivalent wild-type strain ATCC 43816 (27). Reference and MDR clinical strains were cultured in the appropriate liquid broth until OD_600_ = 0.6 and then 15-mL growth medium containing agar were distributed across different plates and allowed to cool. The Etbr was added to the growth medium at concentrations ranging from 0 to 2.5 mg/L to create a cartwheel pattern. A single distinct swab was dipped into each culture and streaked from the center circle to the edge of the plate to avoid straying from the underlying line. The swabbed lines were arranged as follows: the strain reference at 12 o'clock and swabbed strains at o'clock locations of 1.5, 3, 4.5, 6, etc. The EtBr-agar plates were incubated for different times at 37℃ and inspected using an appropriate UV light source. For both the reference and MDR clinical strains, the lowest concentration of EtBr required to cause fluorescence in the swabbed bacterial mass was recorded. The presumed efflux pump activity of an organism increases with the concentration of EtBr required to exhibit fluorescence (28).

### MDR reversal assay

The half-effective concentration (EC_50_) refers to the concentration at which the drug produces 50% of the maximum effect on the corresponding symptoms in the efficacy experiment, which is an important index in the efficacy evaluation (29). To evaluate the bactericidal interactions between antibiotics and PaβN, a checkerboard approach was used along with drug-free blank control groups. Antibiotics at 1/32 to 1 the MIC were added to the wells of 96-well microtiter plates containing approximately 1 × 10^6^ CFU/mL of the inoculum in each well, and the antibiotic concentration was plotted on the *x*-axis, while PaβN was plotted on the *y*-axis of the plate. After 16–18 h of incubation at 37℃, the OD_600_ was measured, and visual readings were used to calculate the MICs. A non-linear regression analysis was used to determine EC_50_ values using GraphPad Prism 9.0 (30).

### Deletion and complementation of *oqxB*

Knockout of *oqxB* was achieved following a strategy adjusted from Datsenko and Wanner. First is the validation of strain resistance. Strains with monoclones were inoculated separately in liquid medium with corresponding antibiotic resistance and grown 37℃ overnight. Homology fragments of *oqxB* (about 500 base pairs upstream and downstream) were amplified by PCR using the primers listed in Table S3. The fragments were then inserted into the plasmid pUC19. The fragments used for constructing the *oqxB* knockout were amplified by template using the sequencing correct *oqxB*-UD-pUC 19 plasmid. The fragments were then ligated into the vector pAaprSacB, transformed into competent cell *E. coli* S17 pir, and coated with apramycin-resistant plates, and positive clones were sent for sequencing. The next step is knockout screening; the GN 172867 strain and *oqxB*-pAprSacB S17λ pir monoclone were inoculated into Luria-Bertani (LB) liquid medium and LB (Apr-containing resistance), respectively; and OD_600_ was grown to about 0.8 for binding transfer experiments. Seven hundred microliters of GN 172867 and *oqxB*-pAprSacB S17 pir were mixed by centrifugation, and the bacteria were incubated in sterilized nitrocellulose filter central LB medium overnight. The bacterial solution was washed with 1-mL LB medium and then diluted with Amp/Apr double antibody plates. The identified positive single-exchange strains were inoculated into LB medium with 10% sucrose, and the poor Apr resistance was selected for identification, and the positive clones were selected for identification with the wild strain. Mutant clones were confirmed by PCR and sequencing. The *oqxB* deleted transformant was designated as GN 172867∆*oqxB*.

The wild-type *oqxB* of GN 172867 was amplified by PCR using primers poqxB-ptac-F/R. The products were cloned into the vector pApr-promoter*oqxB* to generate the recombinant vector p-*oqxB*. p-*oqxB* was confirmed by both Apr selection and sequencing. Then, p-*oqxB* was electrically transformed into GN 172867∆*oqxB*. The positive clone was designated as GN 172867∆*oqxB*/p-*oqxB*. The empty plasmid pApr-promoter*oqxB* was used as a negative control. The primers used are listed in Table S3.

### Molecular modeling docking

ChemDraw 20.0 was used to draw the molecules, PyMOL was used to minimize them, and a mdb file was saved. Target OqxB (PDB ID: 7CZ9) was obtained from the RCSB Protein Data Bank (https://www.rcsb.org) and prepared using AutodockTools software by removing water, adding hydrogen, merging non-polar hydrogen, adding Gasteiger charges, and so forth (31). AutoGrid was used with the following parameters to find the pocket: 0.408 angstrom for the center grid box spacing. Molecular docking was then run following the establishment of a strict filename and the selection of the genetic algorithm with 200 Genetic Algorithm (GA) runs. To assess the potential of the corresponding compounds and examine the interactions between target OqxB and PaβN, the docked conformation with the largest cluster and the lowest binding energy was selected from 200 conformations as the representative binding energy. Following the completion of molecular docking, AutoDockTools software was used to evaluate and PyMOL software was used to visualize the interactions between the docked domain and PaβN.

### Clinical data collection

Clinical data of patients were collected, mainly including the following aspects: demographic characteristics of patients (gender and age), the department where the infection occurred, the source of infection, and clinical manifestations (bacterial resistance and drug sensitivity analysis, antibiotic treatment plan, and anti-infection effect).

### Statistical analysis

Error bars show standard errors of the means for each experiment, which was run in triplicate. The Kruskal–Wallis one-way analysis of variance test and the Student’s *t*-test were used to compute and compare the differences. The following notation denotes statistically significant differences: *P < 0.05, **P < 0.01, ***P < 0.001, and ****P < 0.0001. All graphs were generated using GraphPad Prism 9.0 (GraphPad Inc., San Diego, CA, USA) and Adobe Illustrator CC 2021 (Adobe Systems Inc., USA).

## RESULTS

### Identification of 12 clinical quinolones-resistant *K*. *pneumoniae* strains

**Fig 1:**
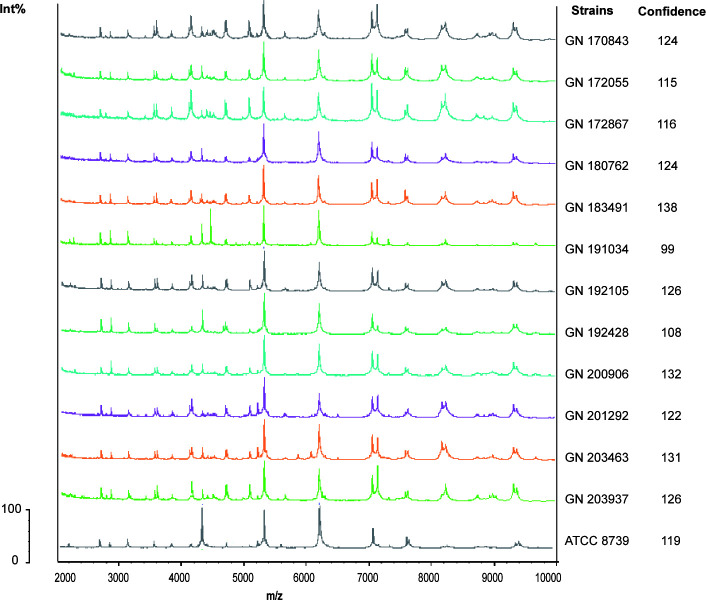
Identification of MDR *K. pneumoniae* clinical isolates. MALDI-TOF mass spectrometric profiles of 12 bacterial isolates. The measured range of 2,000–10,000 Da is displayed. The characteristic mass peaks are predominantly ribosomal proteins. The peak of mass spectrometry of *K. pneumoniae* was 4,365, 5,381, and 7,158 Da. Deviations within 5 Da are considered normal. Computer display of identification results after automatic comparison of the generated spectrum with the MALDI-TOF database. The degree of similarity to the reference spectrum is represented by a score value. Identification results with score values above 25 are considered to be correct for the determination of the respective species.

We identified 12 clinical quinolone-resistant *K. pneumoniae* strains from strains preserved by the Anhui Bacterial Resistance Center using Clin-TOF-II/MS with confidence >100 (Fig. 1). The MICs of the strains against 20 different antibiotics, including quinolones, were investigated using the broth dilution method (Table S1), which identified a quinolone-resistant phenotype in the 12 isolated strains.

**Fig 2:**
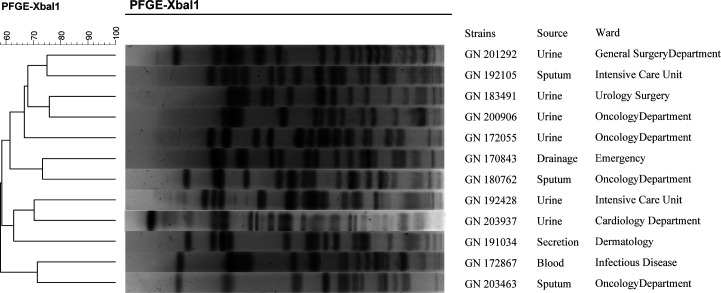
Polymorphism analysis. PFGE cluster analysis of 12 strains of MDR *K. pneumoniae* from different sources. Restriction enzyme *XbaI* was used for genomic DNA digestion to generate restriction fragment length polymorphisms (RFLPs). The ruler indicates percent RFLP similarity. The dendrogram was developed using BioNumerics analysis software. Percent similarities are described by the unweighted pair group method using arithmetic average (UPGMA) method. These experiments were performed at least three times, and representative results are shown.

PFGE was used to analyze polymorphisms in 12 *K*. *pneumoniae* strains, and following the digestion of *XbaI* and processing using BioNumerics software, a tree diagram was produced (Fig. 2). The results showed that the 12 strains of *K. pneumoniae* contained 44 groups of distinct PFGE bands, suggesting that the strains were highly polymorphic. DNA fingerprinting determined that similarity coefficient of these strains, as determined by was less than 80%. As these isolates were obtained from different specimens collected from different patients, they were significantly epidemiologically different.

### Two quinolone-resistant *K*. *pneumoniae* strains with the most and least *oqxB* efflux pump gene expression level were selected

**Fig 3:**
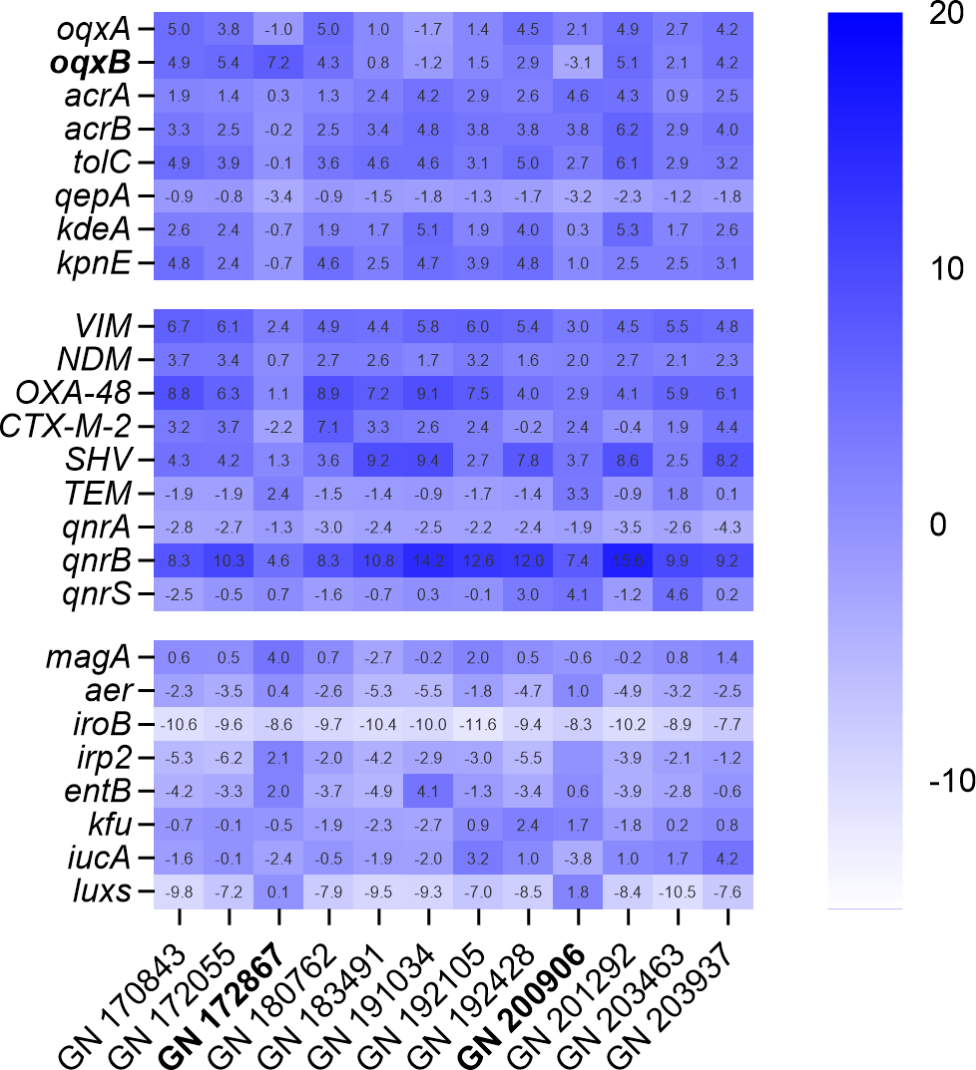
qRT-PCR assessment of the expression of drug resistance-related genes. Log_2_-fold change of the *acrA*, *acrB*, *tolC*, *oqxA*, *oqxB*, *luxS*, *qepA*, *kdeA*, *kpnE*, *iucA*, *VIM*, *NDM*, *OXA-48*, *CTX-M-2*, *SHV*, *TEM*, *qnrA*, *qnrB*, *qnrS*, *magA*, *aer*, *iroB*, *irp2*, *entB*, and *kfu* genes in the 12 *K*. *pneumoniae* strains. ATCC 43816 was used as the reference strain, and *rrsE* was the reference gene. Three independent replicates were tested for each gene.

The emergence of quinolone resistance poses a growing concern to human health, particularly in the context of infections caused by *K. pneumoniae*, and the upregulation of efflux pump systems is a key factor in this resistance (32). The RND superfamily encoding genes include *oqxA*, *oqxB*, *acrA*, *acrB*, *tolC*, and *qepA*. KdeA is an MFS efflux pump-encoding gene. KpnE is an SMR efflux pump-encoding gene, and *VIM*, *NDM*, and OXA-48 are carbapenemase-encoding genes. *CTX-M-2*, *SHV*, and *TEM* are extended-spectrum beta-lactamase (ESBL)-encoding genes, while the plasmid genes *qnrA*, *qnrB*, and *qnrS* encode proteins in the pentapeptide repeat family that protects DNA gyrase and topoisomerase IV from quinolone inhibition, while *magA*, *aer*, *iroB*, *irp2*, *entB*, *kfu*, and *iucA* are virulence genes. The quorum-sensing *luxS* gene has been linked to the regulation of biofilm formation by pathogens. The expression levels of efflux pump-related genes *oqxA*, *oqxB*, *acrA*, *acrB*, *tolC*, *luxS*, *qepA*, *kdeA*, and *kpnE* in these 12 strains were determined using qRT-PCR (Fig. S1) with ATCC 43816 as the control strain and *rrsE* as the reference gene. The qRT-PCR results indicated that the strain GN 172867 exhibited the highest expression of the *oqxB* gene (7.19 ± 0.06 log_2_-fold), while the strain GN 200906 displayed the lowest expression of the *oqxB* gene (−3.11 ± 0.31 log_2_-fold) relative to the reference strain ATCC 43816 (Fig. 3). In addition to *oqxB*, there were significant differences in the expression levels of *qnrB*, *qepA*, *VIM*, *OXA-48*, *CTX-M-2*, *TEM*, *magA*, and *iroB* between GN 172867 and the reference strain ATCC 43816 (Fig. 3). Moreover, significant differences were observed in the expression levels of *acrA*, *acrB*, *tolC*, *qepA*, *VIM*, *OXA-48*, *CTX-M-2*, *SHV*, *TEM*, *qnrB*, *qnrS*, *magA*, *iroB*, and *iucA* between GN 200906 and the reference strain ATCC 43816 (Fig. 3). IroB and luxs are downregulated in all 12 strains. The *qnrB* gene is overexpressed in most strains. However, QnrB provides low-level resistance to quinolones compared to OqxB. Overall, the MIC assays and qRT-PCR results suggested that GN 172867 and GN 200906 had the same phenotype but different genotypes and were selected for further investigation.

Expression levels of the drug efflux pump gene *oqxB* were different in all the clinical strains with multidrug resistance, and the clinical data of GN 172867 and GN 200906 were retrospectively analyzed. Both strains showed similar MIC values for most of the antibiotics tested, including resistance to levofloxacin (LVX) and ciprofloxacin (CIP) and sensitivity to vancomycin and meropenem. The empirical drug regimens after bloodstream infection and the regimens adjusted according to the etiology results were summarized, analyzed, and evaluated as anti-infection regimens (Table 1).

**TABLE 1:** Clinical data of the two patients^*a*^

Strains	Gender	Age (years)	Departments	Source of infection	Specimen	Disease
GN 172867	Male	54	Department of Infectious Diseases	Lung	Blood	Septicemia
GN 200906	Female	53	Department of Radiation Oncology	Urinary system	Urine	Urinary tract infection

Strains	MIC								Therapy	Outcome
	ESBL	CIP	LVX	VAN	MEM	IMP	CLI	CSL		
GN 172867	+	R	R	S	S	NK	NK	R	CTT	Survived
GN 200906	+	R	R	S	S	S	S	R	CLI, LVX, MEM	Survived

^*a*^ CIP, ciprofloxacin; CLI, clindamycin; CSL, cefoperazone sulbactam; CTT, cefotetan; ESBL, extended-spectrum β-lactamase; IMP, imipenem; MEM, meropenem; LVX, levofloxacin; MEM, meropenem; VAN, vancomycin. ESBL (+), resistance to penicillin, cephalosporins, and aztreonam. NK, absence of this drug sensitivity test results; R, resistance to drug susceptibility test results; S, sensitivity to drug sensitivity test results. NA, not available.

### Verifying the efflux pump function in GN 172867 and GN 200906 by combining drug sensitivity test of PaβN and detection with EtBr as indicator

A previous study suggested that, after the addition of EPI PaβN, bacterial isolates with MIC values decreased to 1/4 or less of the original value and were considered as drug efflux pump-positive strains (33). The MICs of LVX and CIP for *K. pneumoniae* strains were determined by the broth microdilution method, which showed that in the presence of PaβN, the MIC of CIP for GN 172867 strain decreased from 256 to 32 mg/L and that of LVX decreased from 128 to 4 mg/L, which was 1/32 times the original value. After adding PaβN, the MIC of CIP for GN 200906 decreased from 256 to 128 mg/L, and the MIC of LVX for GN 200906 decreased from 256 to 32 mg/L, which was 1/8 times the original value (Table 2). Six antibiotics were then used in combination with PaβN and verapamil, respectively. Verapamil was developed as a calcium channel blocker for the treatment of hypertension, but it is also a substrate and inhibitor of P-glycoprotein, a mammalian drug efflux protein, and of transporter protein such as ATP-binding ABC efflux pump (34). Based on the qRT-PCR results, we hypothesized that GN 172867 is a *K. pneumoniae* strain that mediates drug resistance through the overexpression of the OqxB efflux pump. Thus, we expected that verapamil (50 mg/L) would not effectively reverse the resistance of GN 172867 to LVX, CIP, nitrofurantoin (NIT), or tetracycline (TCY). The MICs of LVX, CIP, NIT, and TCY for GN 172867 were reduced by PaβN. A 50-mg/L PaβN effectively reversed the resistance of GN 172867 to LVX, CIP, NIT, and TCY, while 50-mg/L verapamil did not improve antibiotic sensitivity in this strain (Table 3).

**TABLE 2:** CIP and LVX MICs in the absence or presence of PaβN in 2 MDR *K. pneumoniae* isolates^*a*^

Strains	MIC (mg/L)			
	CIP	CIP + PaβN	LVX	LVX + PaβN
GN 172867	256	64	128	8
GN 200906	256	128	256	32

^*a*^ CIP, ciprofloxacin; LVX, levofloxacin; MIC, minimum inhibitory concentration; PaβN, phenylalanine-arginine-β-naphthylamide. The final concentration of PaβN was 50 mg/L.

**TABLE 3:** Susceptibilities of GN 172867 or GN 200906 to those antibiotics, with and without the presence of 50-mg/L PaβN or verapamil^*a*^

Strains	MIC (mg/L)								
	LVX	LVX + PaβN	LVX + verapamil	CIP	CIP + PaβN	CIP + verapamil	NIT	NIT + PaβN	NIT + verapamil
GN 172867	128	8	128	256	64	256	256	16	256
GN 200906	256	32	256	256	128	256	256	16	256

Strains	MIC (mg/L)								
	TCY	TCY + PaβN	TCY + verapamil	GEN	GEN + PaβN	GEN + verapamil	CXM	CXM + PaβN	CXM + verapamil
GN 172867	512	128	512	64	64	64	>1024	>1024	>1024
GN 200906	1024	32	1024	64	64	64	>1024	>1024	>1024

^*a*^ CXM, cefuroxime; GEN, gentamicin; NIT, nitrofurantoin; TCY, tetracycline. The final concentration of PaβN and verapamil was 50 mg/L.

**Fig 4:**
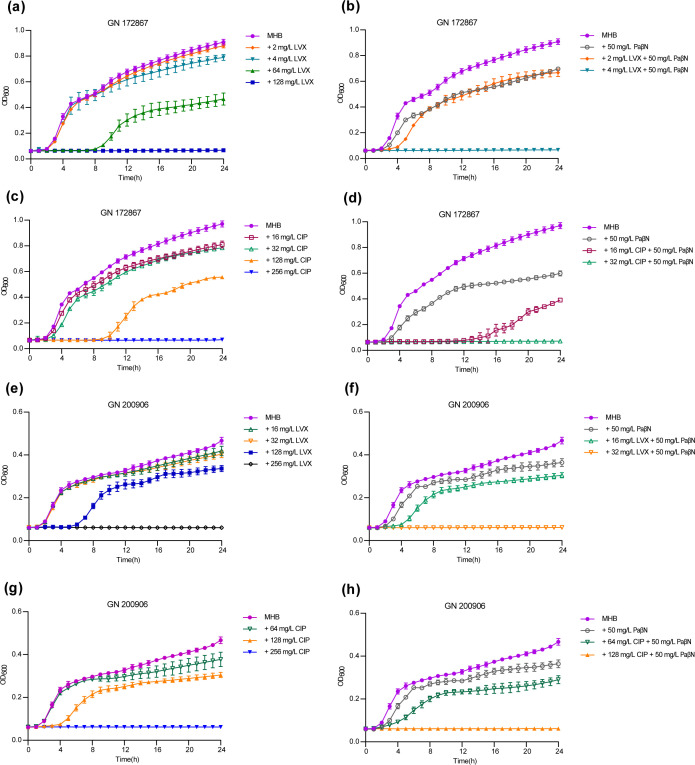
Growth capacity of *K. pneumoniae* strains GN 172867 and GN 200906 under different conditions. Strain GN 172867 was grown in (**a**) LVX, (**b**) LVX + PaβN, (**c**) CIP, and (**d**) CIP + PaβN. Strain GN 200906 was grown in (**e**) LVX, (**f**) LVX + PaβN, (**g**) CIP, and (**h**) CIP + PaβN. Data are the mean OD_600_ values of four independent experiments. OD_600_, optical density at 600 nm. Data are presented as mean ± standard deviation (*n* = 4 biological replicates).

The growth of GN 172867 and GN 200906 strains treated with LVX, LVX + PaβN, CIP, or CIP + PaβN were investigated and showed that the EPI PaβN increased the sensitivity of MDR *K. pneumoniae* strain GN 172867 to quinolones (Fig. 4).

**Fig 5:**
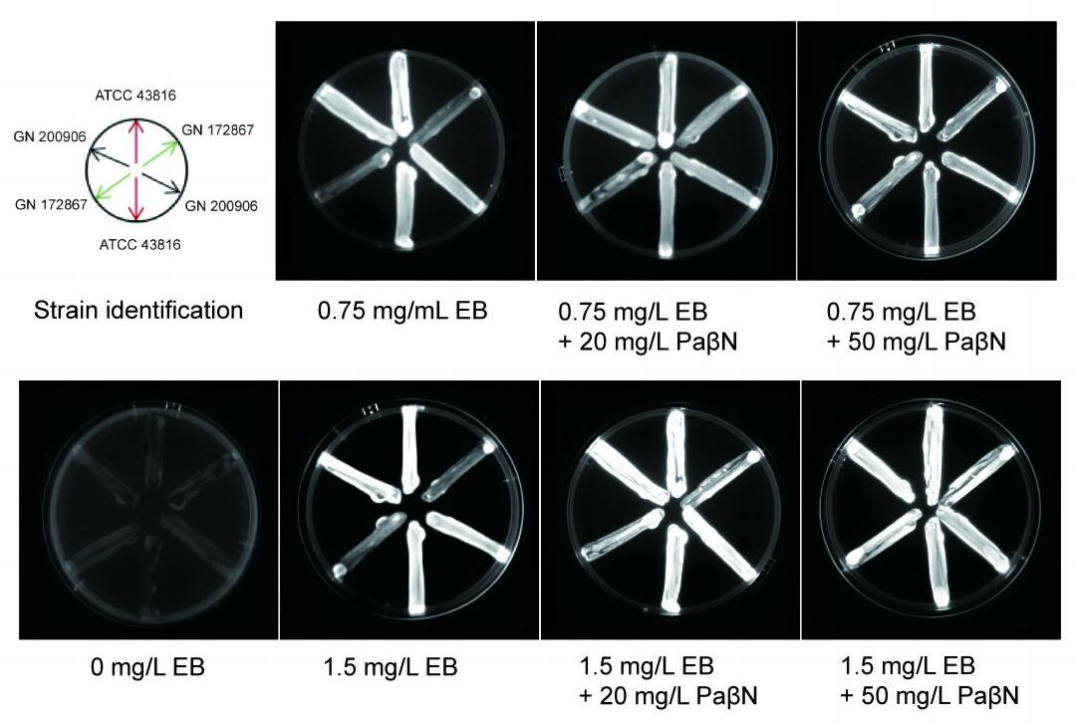
Fluorescence of *K. pneumoniae* strains on agar plates containing increasing concentrations of EtBr and PaβN. Cultures were swabbed in MHA plates containing increasing concentrations of EtBr. Following incubation at 37°C for 6 hours, fluorescence was detected under UV light.

The EtBr-agar cartwheel method was used to phenotypically identify of efflux pump-overexpressing strains. The principle of this method is simple and relies on the ability of the bacteria to expel a fluorescent molecule, such as EtBr, which can act as a substrate for most efflux pumps. In this assay, the higher the concentration of EtBr required to produce fluorescence in the bacterial mass, the greater the efflux capacity of the bacterial cells (27, 28). The results showed that the brightness of GN 172867 was lower than those of ATCC 43816 and GN 200906, which was consistent with the reduced MIC fold determined above after the addition of PaβN (Fig. 5).

The results of qRT-PCR, MIC assays, and the EtBr-agar cartwheel method suggested that the resistance of GN 172867 to quinolones may be due to the overexpression of *oqxB*. Hence, we conducted *oqxB* knockout and complementation experiments to verify this hypothesis.

### The antibiotic activity of against GN 172867 is dependent on PaβN concentration

**Fig 6:**
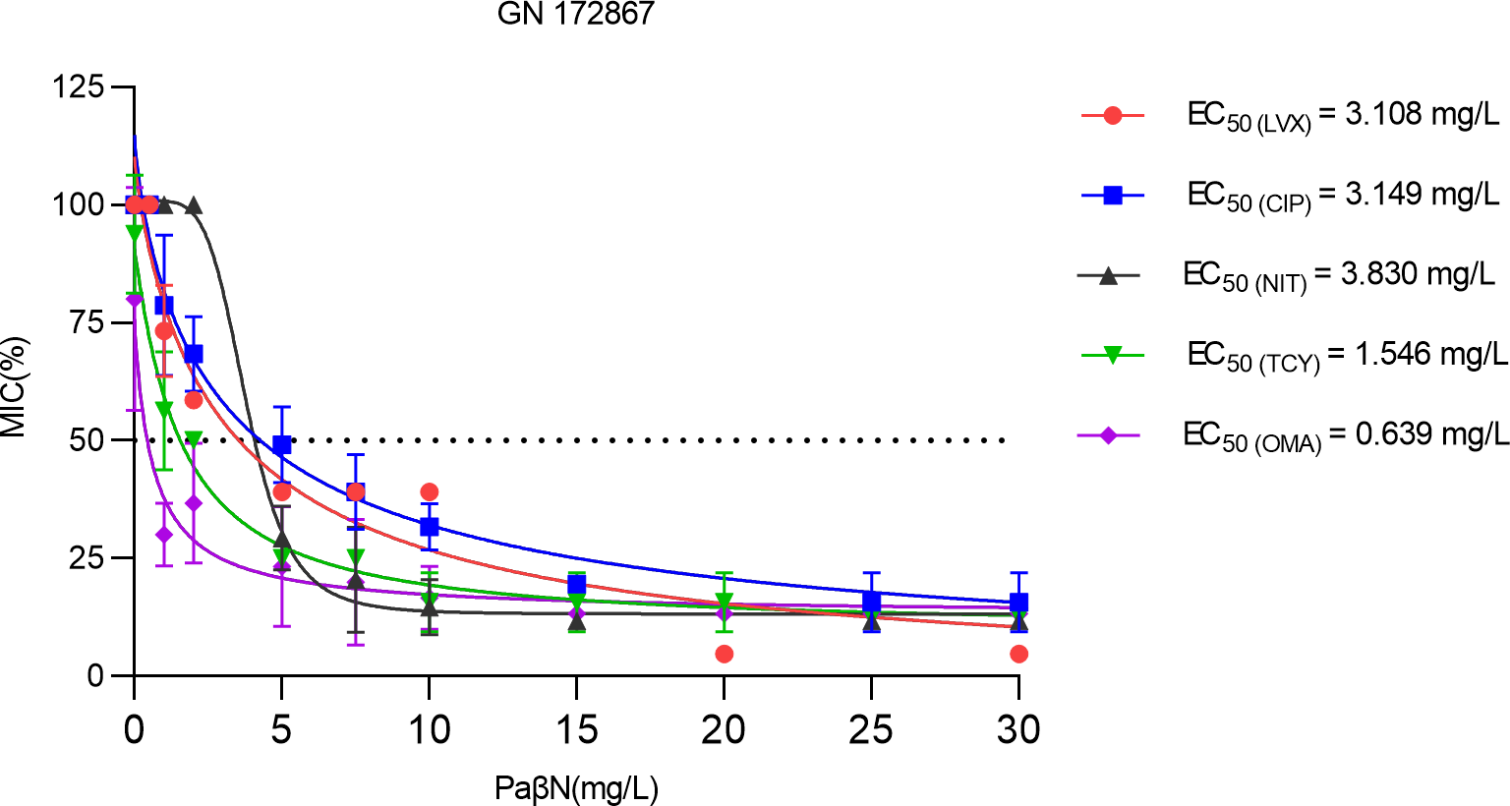
EC_50_ values of PaβN for reversal of LVX, CIP, NIT, TCY, and OMC resistance in *K. pneumoniae* GN 172867. The percentage of the MIC of each antibiotic for *K. pneumoniae* GN 172867 was calculated as follows: [(MIC of each antibiotic at each PaβN concentration/MIC of each antibiotic without PaβN) × 100%)]. EC_50_ refers to the concentration of the PaβN that can reduce the percentage of MIC of each antibiotic for GN 172867 by half. Each data point is presented as the mean of four independent tests.

To evaluate the bactericidal interactions between antibiotics and PaβN, a checkerboard approach was used. The five antibiotics were LVX, CIP, NIT, TCY, and omacycline (OMC). The quinolones, LVX and CIP, are often used to treat pulmonary or urinary tract infections caused by *K. pneumoniae*. NIT, which is a nitrofurans antibiotic, is commonly used in the treatment of urinary tract infection caused by *K. pneumoniae*. OMC, which is a novel TCY drug, is commonly used in the treatment of pulmonary infections caused by *K. pneumoniae*. The compound PaβN was able to reverse LVX, CIP, NIT, TCY, and OMC resistance in *K. pneumoniae* GN 172867 with EC_50_ values of 3.108, 3.149, 3.830, 1.546, and 0.639 mg/L, respectively (Fig. 6). Checkerboard assays showed that the antibacterial activity of LVX, CIP, NIT, TCY, and OMC against GN 172867 was dependent on the concentration of PaβN. Based on these results, PaβN was selected for additional study.

### Efflux pump OqxB plays an important role in quinolone resistance

**Fig 7:**
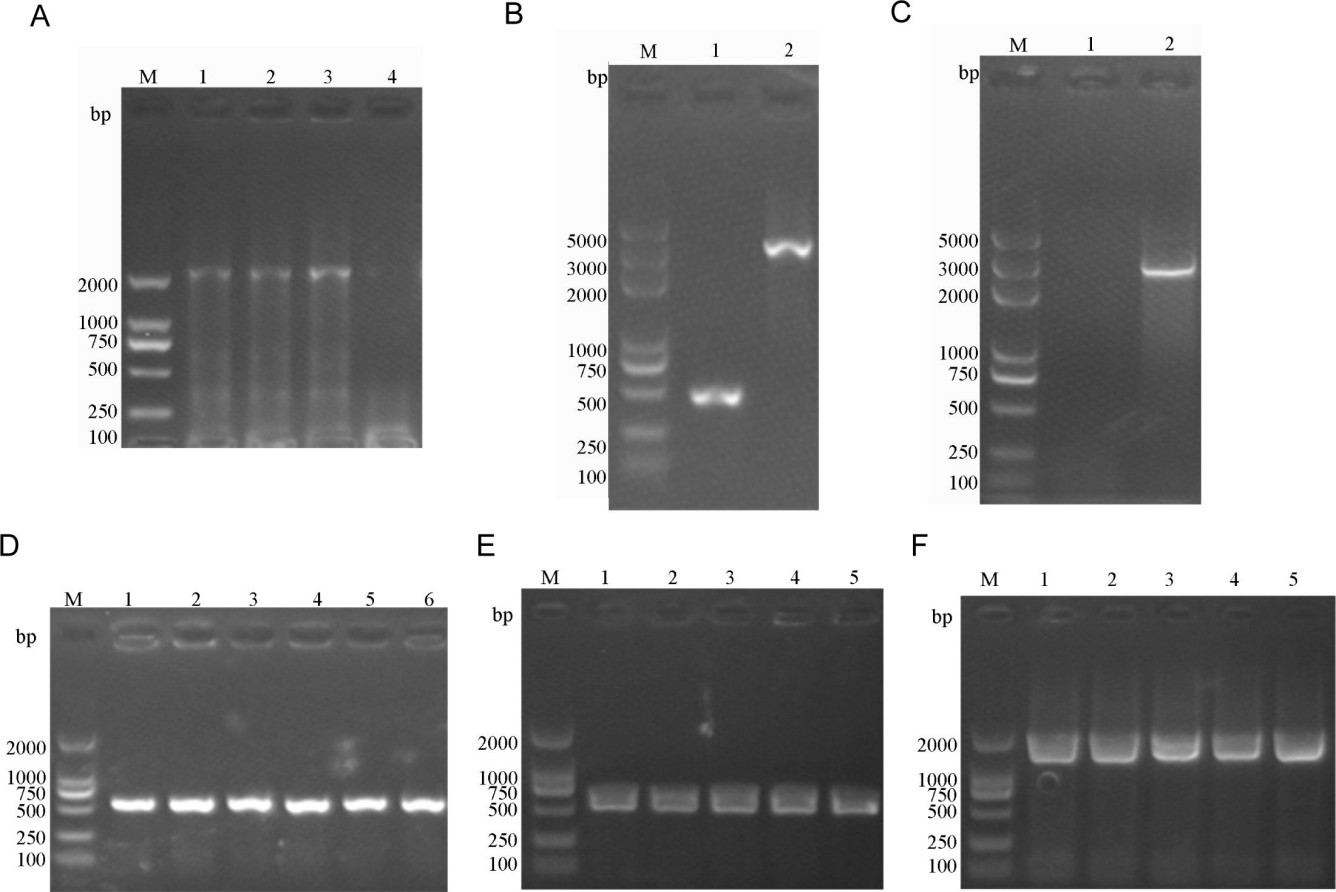
Construction and identification of gene knockout and complementation strains by PCR. Target gene is *oqxB* (3153 bp). Arrows indicate the positions of primers for PCR analyses. (**A**) *oqxB* knockout vector constructs were colonies by PCR. (**B and C**) PCR analysis of the mutant (1) strain and wild (2) strains. Identification of *oqxB* knockout in wild *K. pn* GN 172867. (**D**) Identification of empty plasmid complementation in wild strains. (**E**) Identification of empty plasmid complementation in *oqxB* mutant strains. (**F**) Identification of *oqxB* complementation in *oqxB* mutant strains.

OqxB is a common efflux pump that contributes to antibiotic resistance in *K. pneumoniae*. The *oqxB* gene was expressed in strain GN 172867 but was absent in strain GN 200906 (Fig. S2). To further confirm the role of OqxB, *oqxB* mutant and complementation strains were constructed (designated as GN 172867∆*oqxB* and GN 172867∆*oqxB*/p-*oqxB*; Fig. 7).

The deletion of *oqxB* led to a fourfold decrease in the MIC of LVX (128–32 mg/L), a twofold decrease in the MIC of CIP (256–128 mg/L), and a fourfold decrease in the MIC of NIT (128–32 mg/L; Table 4). The MIC of TCY and OMC showed no significant changes in the *oqxB* mutant strain. After complementation with *oqxB*, the *oqxB* mutant strain had restored resistance against these antibiotics (Table 4). The growth curves are shown in Fig. S3. Plasmids or plasmid-encoded genes did not affect bacterial growth in the absence or presence of antibiotics. We demonstrated that the overexpression of the efflux pump OqxB plays an important role in quinolone resistance, and these findings of this study may help expand our knowledge of quinolone resistance in *K. pneumoniae*.

**TABLE 4:** Results of drug sensitivity test for each strain

Strains	MIC (mg/L)				
	LVX	CIP	NIT	TCY	OMC
GN 172867	128	256	128	256	8
GN 172867∆oqxB	32	128	32	256	8
GN 172867/p-empty	128	256	128	256	8
GN 172867∆oqxB/p-empty	32	128	32	256	8
GN 172867∆oqxB/p-oqxB	128	256	128	256	8
ATCC 43816	0.06	0.06	128	4	2

### *In silico* molecular docking mode between PaβN and OqxB

**Fig 8:**
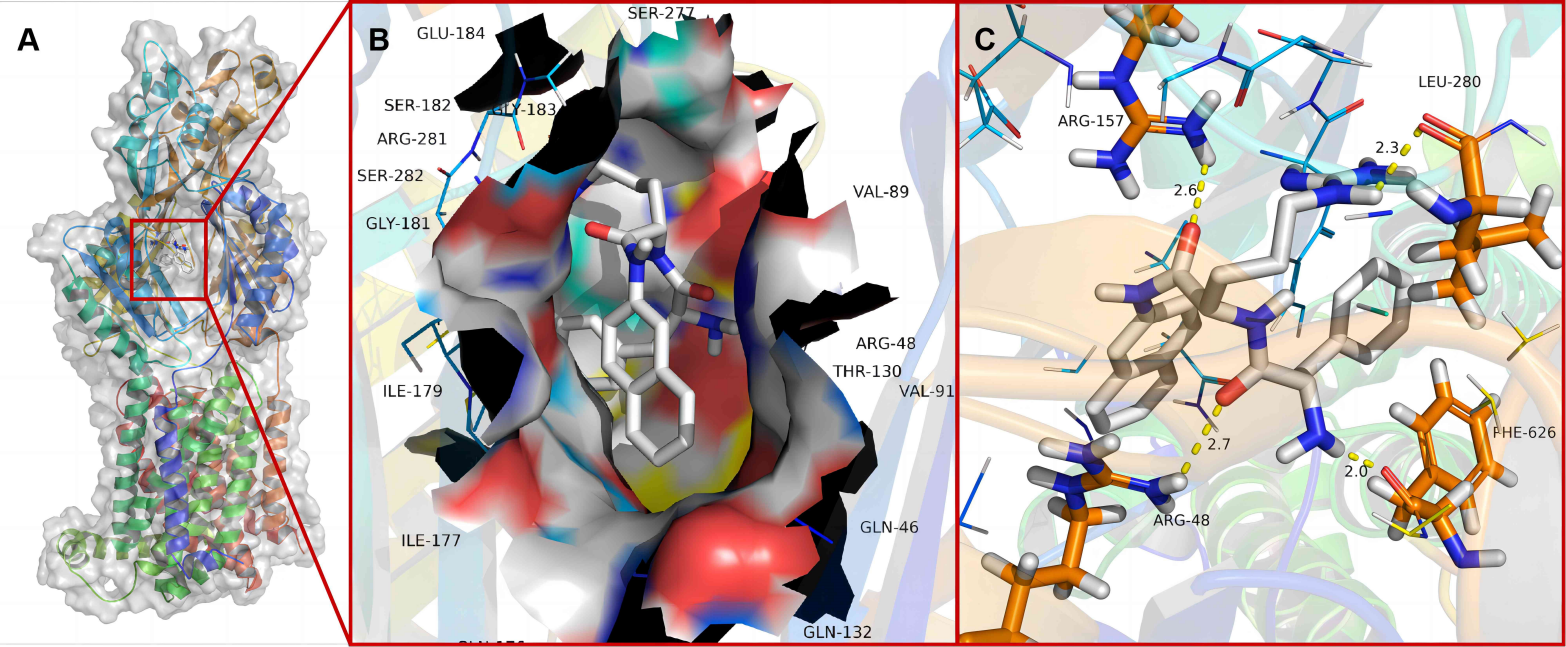
Predicted binding modes of PaβN with OqxB. OqxB structural features. The OqxB structure single protomer with important sub-domains is highlighted with varied colors (**A**). All the predicted docking modes are superposed to illustrate the pattern of PaβN with OqxB substrate-binding pocket (**B**). Molecular docking predicted binding orientations of PaβN (**C**). Inter-molecular hydrogen bond interactions are highlighted with broken lines.

To investigate the binding energy and interactions between compound PaβN and OqxB (PDB code: 7CZ9), we used a molecular docking approach. To create a grid for docking simulations, residues surrounding the DDM1 molecule were considered binding pocket residues. Out of 200 conformations, the docked conformation with the largest cluster and the lowest binding energy was selected as the mode and typical binding energy of the PaβN-OqxB complex. Because of the L-shaped conformation of the line system in PaβN, it was well enveloped by the active pockets of OqxB (Fig. 8A). The PaβN structure satisfied the previously documented need for an extremely potent OqxB inhibitor. Furthermore, the O atoms in the peptide group of PaβN formed two H-bond contacts with ARG 157 and ARG 48 in OqxB, thereby further stabilizing the PaβN-OqxB complex (Fig. 8B). As expected, H atoms of the amidogen and guanidyl in PaβN formed the two H-bond interactions with O atoms of the carbonyl in residues IEU 280 and PHE 626 in OqxB (Fig. 8C). Taken together, these findings provided the structural details required to develop new OqxB inhibitors.

## DISCUSSION

*K. pneumoniae* is a major cause of antimicrobial-resistant healthcare-associated infections, community-acquired liver abscesses, and neonatal sepsis and is associated with intestinal diseases (35). Multidrug efflux pumps, particularly RND family transporters such as OqxB, are critical for antibiotic resistance in Gram-negative bacteria, including intrinsic and acquired resistance to structurally diverse drugs. The OqxB operon was originally described in the plasmid pOLA52, carried by an *E. coli* strain isolated from swine manure (36). Since then, isolates of *K. pneumoniae* from various parts of the world have reported to contain OqxB. Determining the precise function of efflux pumps in quinolone susceptibility is challenging because clinical isolates often have complex resistance backgrounds.

Previous research has demonstrated that the *K. pneumoniae* OqxB efflux pathway confers a higher level of resistance to antibacterial drugs in collaboration with other mechanisms (37); therefore, inhibition of its efflux function or deletion of *oqxB* often increases susceptibility to antibiotics.

Our results indicate that the *K. pneumoniae* strain GN 172867 exhibits a significantly higher expression of the oqxB gene compared to the reference strain ATCC 43816. This overexpression is linked to an increased MICs of quinolones, such as LVX and CIP. The genetic basis for this upregulation remains to be elucidated but may involve mutations, insertions, or regulatory changes affecting the oqxB promoter region or associated regulatory genes. We observed that while the oqxB gene was downregulated in the GN 200906 strain compared to the control strain, the expression levels of other efflux pump genes, including acrA, acrB, and tolC, were slightly upregulated. This suggests that the effect of the EPI PaβN on the MICs of LVX and CIP for the GN 200906 strain is likely due to the combined action of the downregulation of oqxB and the upregulation of other efflux pump genes. This finding underscores the complexity of antibiotic resistance and the potential for EPIs to modulate the effectiveness of antibiotics. The *qnrB* gene is overexpressed in most strains. However, QnrB provides low-level resistance to quinolones compared to OqxB. We acknowledge that due to the small number of clinical *K. pneumoniae* strains (12 strains) studied in our study, there are certain limitations in interpreting the experimental results. However, this does not affect the subject of this study.

The identification of the OqxB efflux pump as a key player in quinolone resistance has significant implications for the development of new antimicrobial agents. Understanding the specific genetic and environmental factors that lead to its overexpression could guide the design of inhibitors that can block the efflux pump, thereby restoring the effectiveness of existing quinolones. Our study also explored the use of PaβN, an EPI, to reverse the resistance phenotype. The successful reduction in MIC values for quinolones in the presence of PaβN suggests a potential therapeutic strategy. This approach could be further optimized by combining PaβN with existing antibiotics to enhance their efficacy against resistant strains.

From a public health perspective, our findings highlight the need for continuous surveillance of antibiotic resistance mechanisms, including the monitoring of efflux pump expression levels in clinical isolates. This information is crucial for guiding empirical therapy and for the development of local and national antibiotic stewardship programs.

While our study provides valuable insights into the role of OqxB in quinolone resistance, further research is needed to fully elucidate the genetic and environmental factors influencing its expression. Additionally, the development of novel inhibitors targeting OqxB requires more in-depth investigation, including *in vitro* and *in vivo* studies to assess their efficacy and safety.

In summary, this research advances our knowledge of the function of the OqxB transporter in *K. pneumoniae* and offers motivation for tackling the quinolone resistance challenge. Therefore, the role of OqxB as a multidrug efflux pump in *K. pneumoniae* requires further investigation.

### Conclusion

In conclusion, the discovery of the *K. pneumoniae* GN 172867 strain will contribute to a better understanding of the role of the OqxB transporter in *K. pneumoniae* and provide inspiration for addressing the threat posed by quinolone resistance.

## Data Availability

The data sets used and analyzed during the current study are available from the corresponding author upon reasonable request.

## SUPPLEMENTAL MATERIAL

Supplemental figures and tables spectrum.00122-24-s0001.doc Fig. S1-S3; Tables S1-S3.

ASM does not own the copyrights to Supplemental Material that may be linked to, or accessed through, an article. The authors have granted ASM a non-exclusive, world-wide license to publish the Supplemental Material files. Please contact the corresponding author directly for reuse.
