# Supplementary material for: Discovery of clinical isolation of drug-resistant Klebsiella pneumoniae with overexpression of OqxB efflux pump as the decisive drug resistance factor
Source: Microbiol Spectr. 2024 Aug 16;12(10):e00122-24. doi: 10.1128/spectrum.00122-24 (PMC11448435; doi:10.1128/spectrum.00122-24)
Supplement: Supplemental figures and tables — Fig. S1-S3; Tables S1-S3. [file spectrum.00122-24-s0001.doc]

ORIGINAL RESEARCH

Ping Tian et al

# Discovery of clinical isolation of drug-resistant *Klebsiella pneumoniae* with overexpression of OqxB efflux pump as the decisive drug resistance factor

Ping Tian ^1,2*^

Ming-Juan Guo ^1,2,4*^

Qing-Qing Li ^1,2^

Xu-Feng Li ^1,2^

Xiao-Qiang Liu ^1,2^

Qin-Xiang Kong ^3^

Hui Zhang ^1^

Yi Yang ^1,2^

Yan-Yan Liu ^1,2^

Liang Yu ^1,2^

Jia-Bin Li ^1,2,†^

Ya-Sheng Li ^1,2,†^

^1^Department of Infectious Diseases & Anhui Center for Surveillance of Bacterial Resistance, The First Affiliated Hospital of Anhui Medical University, Hefei, 230022, China; ^2^Anhui Province Key Laboratory of Infectious Diseases & Institute of Bacterial Resistance, Anhui Medical University, Hefei, 230022, China; ^3^Department of Infectious Diseases, Chaohu Hospital of Anhui Medical University, Hefei, China; ^4^Department of Hepatology, The First Affiliated Hospital of Jilin University, Changchun, 130021, China

* The authors contributed equally to this study.

^†^ Correspondence: Jia-Bin Li; Ya-Sheng Li

Department of Infectious Diseases, the First Affiliated Hospital of Anhui Medical University, Jixi Road 218, Hefei, Anhui, 230022, People's Republic of China

Tel +86-551-62922713

Fax +86-551-62922281

1. mail [lijiabin@ahmu.edu.cn](mailto:lijiabin@ahmu.edu.cn); liyasheng@ahmu.edu.cn

**Table Legends**

**Table S1.** Antimicrobial susceptibility for the strains used in this study

**Table S2.** Primers used in this study

**Table S3.** Primers used for *oqxB* deletion and complementation

**Figure Legends**

**Figure S1.** qRT-PCR assessment of the expression of drug resistance-related genes. Log2-fold change of the *acrA, acrB, tolC, oqxA, oqxB, luxS, qepA, kdeA, kpnE, iucA, VIM, NDM, OXA-48, CTX-M-2, SHV, TEM, qnrA, qnrB, qnrS, magA, aer, iroB, irp2, entB,* and *kfu* genes in the 12 *K. pneumoniae* strain. ATCC 43816 was used as the reference strain and *rrsE* was the reference gene.

**Figure S2.** PCR analysis of the *oqxB* gene of strain (1) GN 200906 and strain (2) GN 172867.

**Figure S3.** Growth capacity of *K. pneumoniae* strains GN 172867, GN 172867∆*oqxB*, GN 172867/p-empty, GN 172867∆*oqxB*/p-empty, and GN 172867∆*oqxB*/p-*oqxB* under different conditions. Data are the mean OD_600_ values of three independent experiments. OD_600_, optical density at 600 nm. Data are presented as mean ± standard deviation (n = 3 biological replicates).

Table S1. Antimicrobial susceptibility for the strains used in this study

|  | Quinolones | | Nitrofurans | Tetracyclines | | Penicillins | | Cephalosporins alkene | | Monocyclic lactam |
| --- | --- | --- | --- | --- | --- | --- | --- | --- | --- | --- |
| Strains | CIP | LVX | NIT | TCY | DOX | AMP | PIP | CRO | CXM | ATM |
| GN 170843 | 1024 | 128 | 512 | >128 | 32 | >256 | >1024 | >32 | >256 | 64 |
| GN 172055 | 256 | 128 | 512 | 128 | 32 | >256 | 512 | >32 | >256 | 16 |
| GN 172867 | 256 | 128 | 1024 | >128 | 32 | >256 | >1024 | >32 | >256 | 64 |
| GN 180762 | 256 | 128 | 1024 | 64 | 8 | >256 | >1024 | >32 | >256 | 128 |
| GN 183491 | 256 | 256 | 512 | >128 | 128 | >256 | 512 | 32 | 256 | 4 |
| GN 191034 | 256 | 128 | 256 | >128 | 64 | >256 | 1024 | >32 | >256 | 64 |
| GN 192105 | 256 | 256 | 128 | >128 | 32 | >256 | >1024 | >32 | >256 | >128 |
| GN 192428 | 256 | 128 | 128 | >128 | 64 | >256 | >1024 | >32 | >256 | 128 |
| GN 200906 | 256 | 256 | 256 | >128 | 64 | >256 | 512 | >32 | >256 | 64 |
| GN 201292 | 256 | >256 | 128 | >128 | 128 | >256 | 1024 | >32 | >256 | 128 |
| GN 203463 | 256 | 128 | 256 | 32 | 64 | >256 | >1024 | >32 | >256 | 128 |
| GN 203937 | 256 | 128 | 128 | >128 | 128 | >256 | >1024 | >32 | >256 | 64 |
| ATCC 43816 | 0.06 | 0.13 | 64 | 4 | 2 | 64 | 8 | ≤0.06 | 8 | ≤0.25 |

|  | Carbapenems | | Lipopeptides | Aminoglycosides | | Folic acid metabolic pathway inhibitors | | Styrene acrylic alcohols | Fosfomycin class |
| --- | --- | --- | --- | --- | --- | --- | --- | --- | --- |
| Strains | IMP | MEM | COL | GEN | AMK | SXT | TMP | CHL | FOS |
| GN 170843 | 1 | ≤0.06 | 1 | >128 | >512 | >32 | >128 | >256 | >2048 |
| GN 172055 | 1 | ≤0.06 | 1 | 2 | 2 | >32 | >128 | >256 | 64 |
| GN 172867 | 1 | ≤0.06 | 1 | 64 | 2 | >32 | >128 | >256 | 2048 |
| GN 180762 | 1 | ≤0.06 | 1 | >128 | >512 | >32 | >128 | 64 | 256 |
| GN 183491 | 0.5 | ≤0.06 | 1 | 1 | 2 | >32 | >128 | 64 | 512 |
| GN 191034 | 2 | ≤0.06 | >32 | 128 | >512 | >32 | >128 | >256 | 64 |
| GN 192105 | >32 | 16 | 1 | >128 | >512 | >32 | >128 | 64 | 32 |
| GN 192428 | 2 | ≤0.13 | 1 | 128 | 4 | >32 | >128 | >256 | 256 |
| GN 200906 | 0.5 | 0.13 | 0.5 | 64 | 1 | >32 | >128 | >256 | 32 |
| GN 201292 | 4 | ≤0.06 | 1 | 32 | 4 | >32 | >128 | >256 | 64 |
| GN 203463 | 2 | 0.06 | 0.5 | 8 | 4 | >32 | >128 | >256 | 128 |
| GN 203937 | 0.5 | ≤0.06 | 1 | 64 | 1 | >32 | >128 | >256 | 128 |
| ATCC 43816 | 0.5 | ≤0.06 | 0.5 | 0.5 | ≤1 | 0.25 | 2 | 8 | 128 |

**Notes:** MIC, minimum inhibitory concentration; CIP, ciprofloxacin (the range of ciprofloxacin concentrations used in MIC test was 0.016 - 8 mg/L); LVX, levofloxacin (0.0313 - 16 mg/L); NIT, nitrofurantoin (2 - 1024 mg/L); TCY, tetracycline (0.5 - 128 mg/L); DOX, doxycycline (0.25 - 128 mg/L); AMP, ampicillin (0.5 - 256 mg/L); PIP, piperacillin (1 - 1024 mg/L); CRO, ceftriaxone (0.125 - 32 mg/L); CXM, cefuroxime (0.5 - 256 mg/L); CFP, cefoperazone (1 - 512 mg/L); ATM, aztreonam (0.25 - 128 mg/L); IMP, imipenem(0.125 - 32 mg/L); MEM, meropenem(0.125 - 32 mg/L); COL, colistin (0.125 - 16 mg/L); GEN, gentamicin (0.25 - 128 mg/L); AMK, amikacin (0.125 - 16 mg/L); SXT, trimethoprim-sulfamethoxazole (0.125 - 32 mg/L); TMP, trimethoprim (0.5 - 128 mg/L); CHL, chloramphenicol (0.5 - 256 mg/L); FOS, fosfomycin (4 - 2048 mg/L).

Table S2. Primers used in this study

| **Gene** | **Primer** | **Sequence (5' to 3')** |
| --- | --- | --- |
| *rrsE* | *rrsE*-F | TTGACGTTACCCGCAGAAGAA |
|  | *rrsE*-R | GCTTGCACCCTCCGTATTACC |
| *acrA* | *acrA*-F | ATGTGACGATAAACCGGCTC |
|  | *acrA*-R | CTGGCAGTTCGGTGGTTATT |
| *tolC* | *tolC*-F | CTACAAACAGGCGGTGGTCT |
|  | *tolC*-R | TGTTCAGCTCGTTGATCAGG |
| *acrB* | *acrB*-F | CAATACGGAAGAGTTTGGCA |
|  | *acrB*-R | CAGACGAACCTGGGAACC |
| *oqxA* | *oqxA*-F | GCGTCTCGGGATACATTGAT |
|  | *oqxA*-R | GGCGAGGTTTTGATAGTGGA |
| *oqxB* | *oqxB*-F | CTGGGCTTCTCGCTGAATAC |
|  | *oqxB*-R | CAGGTACACCGCAAACACTG |
| *kdeA* | *kdeA*-F | GTTGTTCCCGTTATGTCTGGTGC |
|  | *kdeA*-R | CCAGCAGCCACTGTAAAAACATGC |
| *kpnE* | *kpnE*-F | ATTGCTGAAATTACCGGCAC |
|  | *kpnE*-R | AAATACCGATCCCTTCCCAC |
| *iucA* | *iucA*-F | CTCAAGCTTAGTGCTTCCTGAATGCCT |
|  | *iucA*-R | CTCGGATCCTATGAGTCACCTGGTCAC |
| *qepA* | *qepA*-F | GCCGGTGATGCTGCTGA |
|  | *qepA*-R | CAGRAACAGCGCSCCSA |
| *luxS* | *luxS*-F | AGTGATGCCGGAACGCGG |
|  | *luxS*-R | CGGTGTACCAATCAGGCTC |
| *VIM* | *VIM*-F | GTTTGGTCGCATATCGCAAC |
|  | VIM-R | AATGCGCAGCACCAGGATAG |
| *NDM* | *NDM*-F | GCAGCTTGTCGGCCATGCGGGC |
|  | *NDM*-R | GGTCGCGAAGCTGAGCACCGCAT |
| *OXA-48* | *OXA-48*-F | GCGTGGTTAAGGATGAACAC |
|  | *OXA-48*-R | CATCAAGTTCAACCCAACCG |
| *CTX-M-2* | *CTX-M-2*-F | CGTTAACGGCACGATGAC |
|  | *CTX-M-2*-R | CGATATCGTTGGTGGTRCCAT |
| *SHV* | *SHV*-F | AGCCGCTTGAGCAAATTAAAC |
|  | *SHV*-R | ATCCCGCAGATAAATCACCAC |
| *TEM* | *TEM*-F | CATTTCCGTGTCGCCCTTATTC |
|  | *TEM*-R | CTCAGTGCTCTACAGAAAACC |
| *qnrA* | *qnrA*-F | AGAGGATTTCTCACGCCAGG |
|  | *qnrA*-R | GCAGCACTATKACTCCCAAGG |
| *qnrB* | *qnrB*-F | GGMATHGAAATTCGCCACTG |
|  | *qnrB*-R | TTTGCYGYYCGCCAGTCGAA |
| *qnrS* | *qnrS*-F | GCAAGTTCATTGAACAGGCT |
|  | *qnrS*-R | TCTAAACCGTCGAGTTCGGCG |
| *magA* | *magA*-F | GGTGCTCTTTACATCATTGC |
|  | *magA*-R | GCAATGGCCATTTGCGTTAG |
| *aer* | *aer*-F | GCATAGGCGGATACGAACAT |
|  | *aer*-R | CACAGGGCAATTGCTTACCT |
| *iroB* | *iroB*-F | ATCTCATCATCTACCCTCCGCTC |
|  | *iroB*-R | GGTTCGCCGTCGTTTTCAA |
| *irp2* | *irp2*-F | GCATTTTCCGTATCGCTCT |
|  | *irp2*-R | GCTTCATAACCTGCCTGATG |
| *entB* | *entB*-F | GATGAAGACGATACCGTGC |
|  | *entB*-R | TTTCGTGTGGCCAGTGACTC |
| *kfu* | *kfu*-F | GAAGTGACGCTGTTTCTGGC |
|  | *kfu*-R | TTTCGTGTGGCCAGTGACTC |

Table S3. Primers used for *oqxB* deletion and complementation

| Primer | Sequence (5' to 3') |
| --- | --- |
| GN*oqxB*-19-F | TGTAAAACGACGGCCAGTCTTCGCCCTCGCGTCTCGGGATA |
| GN*oqxB*-up-R | TTTGTTATCTGCTGCAGGGTCTTGGATTCTCTGATTAGGG |
| GN*oqxB*-down-F | AATCAGAGAATCCAAGACCCTGCAGCAGATAACAAAAAAA |
| GN*oqxB*-19-R | CTATGACCATGATTACGCCTATCTGAAAGCGTTCCGCGAT |
| GN*oqxB*-JD-F | GATAAAGATGGTAAAGCACAGCG |
| GN*oqxB*-JD-R | AACGCCTGCTGGTACTTCAAATC |
| GN*oqxB*-F | TGAACTGCATGaattcccCTTCGCCCTCGCGTCTCGGGATA |
| GN*oqxB*-R | cgatatcgagctctcccTATCTGAAAGCGTTCCGCGAT |
| GN*oqxB*-ter-F | TCGCTATCCCGCTGCTGCCGGTG |
| GN*oqxB*-ter-R | ATGGTCCCGGCGATAAAGGCGAT |
| p*oqxB*-ptac-F | AGTCAATAAACCGGTGGGATCGCGGTTTTTTATCTCCCCC |
| p*oqxB*-ptac-R | CGACGGTATCGATGGATCCTATTTTTATTCCGGTAAATGTA |
| ptac-*oqxB* promoter-F | TTTACCGGAATAAAAATAGGATCCATCGATACCGTCGACAG |
| ptac-*oqxB* promoter-R | GATAAAAAACCGCGATCCCACCGGTTTATTGACTACCGGA |
| *oqxB*-F | TTACCGGAATAAAAATAATGGACTTTTCCCGCTTTTTTAT |
| *oqxB*-R | GTGGTGATGGTGATGGTGGGCGGGCAGATCCTCCTGGACCG |
| papr-*oqxB*-F | CATCACCATCACCACTAGGGATCCATCGATACCGTCGACAG |
| papr-*oqxB*-R | AGCGGGAAAAGTCCATTATTTTTATTCCGGTAAATGTAGC |
| *oqxB*-cx-F | CAGAAGCAGTCTCCGACGCTGA |
| *oqxB*-cx-R | CGATTTTGGCGTTGATCTCCGCGT |
| Ptac-JD-F | gaagccggcggcacctcgctaac |
| M13-R(pUC) | AGCGGATAACAATTTCACACAGG |


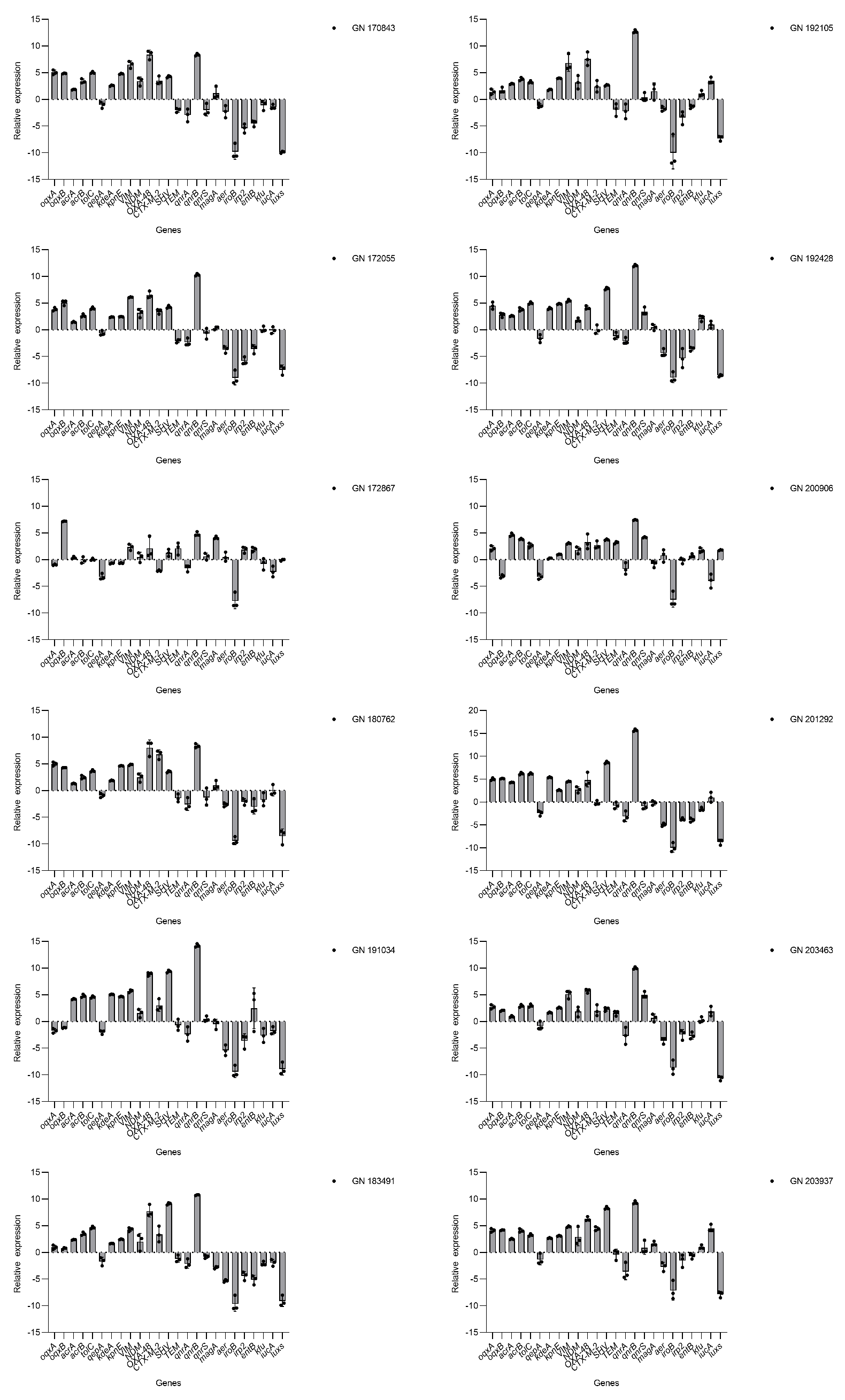


**Figure S1.** qRT-PCR assessment of the expression of drug resistance-related genes. Log2-fold change of the *acrA, acrB, tolC, oqxA, oqxB, luxS, qepA, kdeA, kpnE, iucA, VIM, NDM, OXA-48, CTX-M-2, SHV, TEM, qnrA, qnrB, qnrS, magA, aer, iroB, irp2, entB,* and *kfu* genes in the 12 *K. pneumoniae* strain. ATCC 43816 was used as the reference strain and *rrsE* was the reference gene.


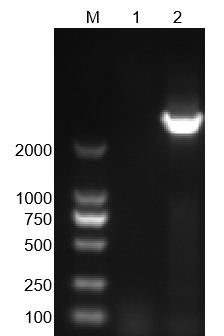


**Figure S2.** PCR analysis of the *oqxB* gene of strain (1) GN 200906 and strain (2) GN 172867.


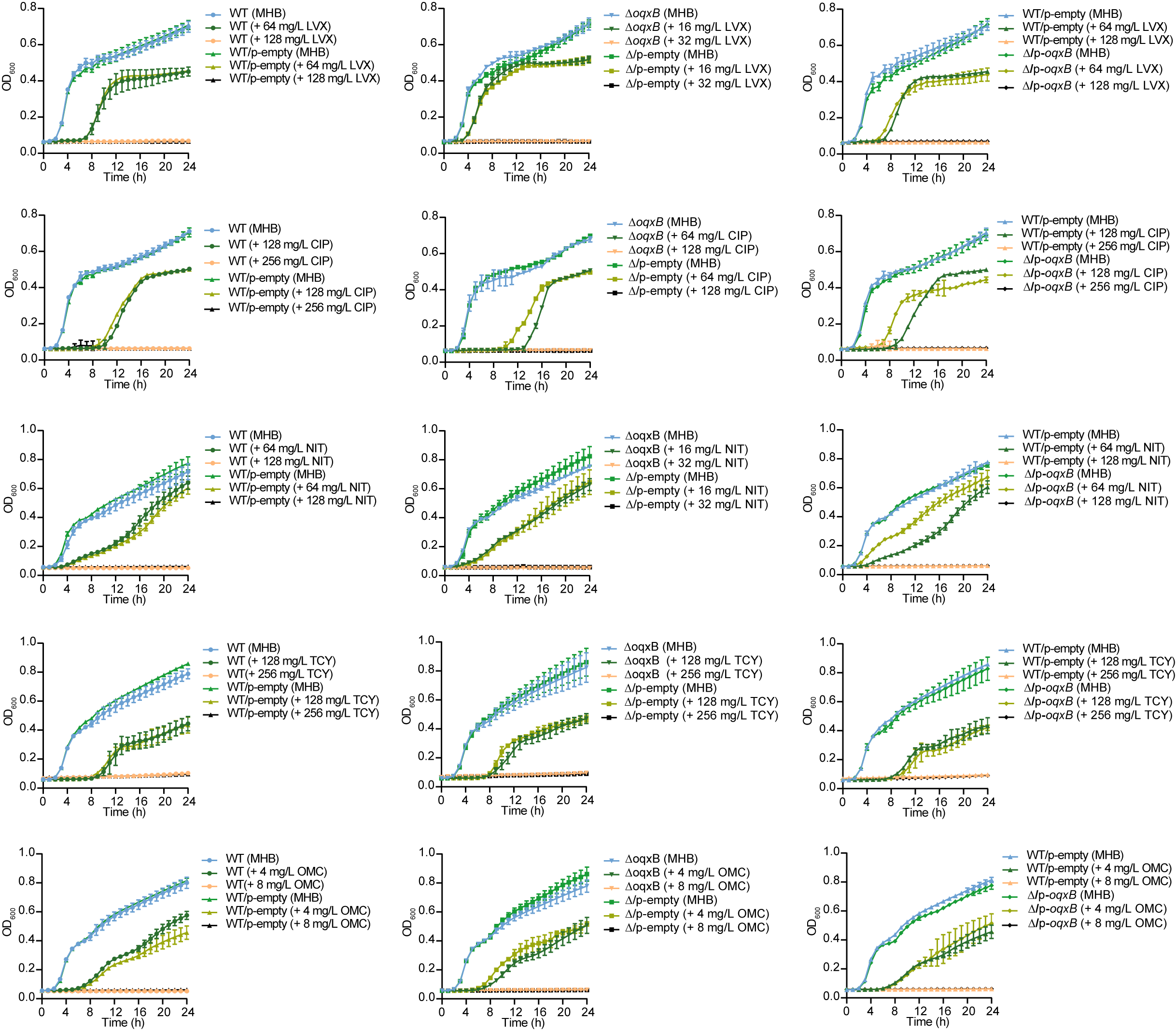


**Figure S3.** Growth capacity of *K. pneumoniae* strains GN 172867, GN 172867∆*oqxB*, GN 172867/p-empty, GN 172867∆*oqxB*/p-empty, and GN 172867∆*oqxB*/p-*oqxB* under different conditions. Data are the mean OD_600_ values of three independent experiments. OD_600_, optical density at 600 nm. Data are presented as mean ± standard deviation (n = 3 biological replicates).
